# Independent Journalism

**Published:** 1898-07

**Authors:** William H. Potter

**Affiliations:** Boston, Mass.


### INDEPENDENT JOURNALISM.[1]

BY WILLIAM H. POTTER, D.M.D., BOSTON, MASS.

In considering the literature of our profession which has appeared during the last ten years, the small number of published books is a matter of note. It would probably be safe to say that in the course of a given year not more than one or two books of importance have made their appearance. This lack of published books does not necessarily indicate that matters of professional interest fail to be considered, or that the results of scientific work fail to be put on record. It simply means that the profession has not yet accumulated sufficient material of an accredited sort that is worthy to assume the permanent form which a published book gives. Meanwhile, to assist the development of the profession and record its progress, a kind of literature is produced having a fresher and less rigid form than is possible with books. This form of literature is the dental journal. What is a journal? To an individual a journal is a diary which recounts all that passes during the day that is of peculiar interest to the one who keeps it. It is a record of the inner thoughts and life of the mind and soul, and a record of such passing events in the outer world as bear in a special way upon the individual. It is often the only honest and complete record which a man may make of himself. To a business concern the journal is the book in which information from all departments of the business is day by day assembled and brought into orderly arrangement and prepared for distribution into proper accounts. It reveals the intimate life of the concern; it records its income and outgo, its adjustment of losses, and its gains. With a ship, the journal is the log; it records every incident of importance in the ship's journey, from the time it leaves a port until it is safely moored at its destination. We find how important the log is to the ship when, in case of murder or mutiny upon the high seas, the log is resorted to as evidence, and its reading gives a minute recital of the intimate life of the ship. The dental journal is as intimately connected with the life of the profession as is the diary with the life of the individual, the journal of the business-house with its life and success, or the log with the progress of a sea-voyage. In order to determine under what auspices dental journals are conducted, I have addressed letters of inquiry to representative men both abroad and

---

[1] Read before The New York Institute of Stomatology.

at home. From Berlin I learn that the dental journals of Germany can be described as follows:

1. The principal journal is the *Deutsche Monatsschrift für Zahnheilkunde. Organ des Central-Vereins-deutscher Zahnärzte.* Edited by Parreidt. Published by Arthur Felix, in Leipsic. This is entirely independent of any dental depot.

2. *Journal für Zahnheilkunde* (weekly). Edited by Erich Richter. An independent journal.

3. *Odontologische Blätter Zahnärztliches Wochenblatt.* Edited by Albrecht. Published by the dental firm of Simonis.

4. *Zahnärtzliche Rundschau.* Edited by Zahnarzt Arthur Bornstein. An independent journal.

5. The *Dental Quarterly* (in German). Published by Messrs. Ash & Sons, London.

In Austria the principal journal is the *Oesterreichisch-Ungarische Vierteljahrschrift für Zahnheilkunde.* Edited by the firm of Julius Weiss. This is a very scientific journal, and its articles are said to be in no way influenced or dependent upon the firm which publishes it.

The English journals can be summarized as follows:

1. The *Journal of the British Dental Association.* Edited by a dental surgeon who has no connection with any manufacturer. Its contents consist of an editorial article, association intelligence, original communications, legal news, reports of societies, clinical reports from hospital and private practice, correspondence, new inventions. Manufacturers take no place, except in the advertisements, for which they pay at a fixed rate. This journal is financially edited by the British Dental Association, one-third of each annual subscription of a guinea being credited to the journal account. The journal appears on the fifteenth of every month.

2. The *British Journal of Dental Science.* The oldest British journal, established in 1856 by Mr. Fox, the dentist, and continued by his partner. Published fortnightly, and having a large circulation. It is a free lance, being the organ of no learned society or manufacturer.

3. The *Transactions of the Odontological Society* is a journal entirely devoted to that society's proceedings, and is perhaps more highly scientific than the others.

4. The *Dental Record.* A monthly journal, published by the Dental Manufacturing Company.

5. The *Dental Quarterly.* Published by Messrs. Ash & Sons.

A list of the dental journals of the United States has been carefully compiled, and can be summarized as follows: Trade journals, —that is, journals published primarily in the interest of manufacturing concerns, and secondarily to furnish literature to dentists,—fifteen; professional journals,—that is, journals published solely in the interest of the profession,—two; doubtful journals,—journals whose backing is not apparent and which are suspected of belonging to the class of trade journals,—three.

From a consideration of the above lists, it will appear that in Germany the professional journals have a strong position and that the trade journals are of much less importance. In England we find professional journalism taking the lead over trade journalism; but in the United States we find the trade journals far outnumbering the professional journals. The points to be kept in mind in any discussion of journalism are, first, the fact that we express ourselves mainly in journals and not in books; second, that the journals record the intimate life of the professional work; and, third, that by far the larger number of journals in the United States are published under a form of paternalism, which means a divided interest.

The question naturally arises, Should we intrust that which so largely affects our welfare to the custody of those who have other things to care for besides that which concerns us?—to those who wish primarily to dispose of articles of merchandise, and who use the dental journals as a means of advertising their wares? I do not wish to be understood as speaking in a slighting way of trade journals; they constitute a method of conducting a business enterprise, and, looked at from the view of the dental dealer, they are perfectly legitimate, and in some cases are ably conducted. In the early days of the dental profession, when there was little combination among members in the form of societies, when the resources of the profession were few, it is probable that the trade journal, with its ability to supply necessary capital, conferred a benefit on the profession by recording matters of vital importance to its growth. Having once assumed this function, and having found it profitable so to do, the trade journal has accustomed the profession to a form of paternalism. The motives of the trade journal are not bad, but they are divided. There is a desire to serve our professional life, but that desire is controlled by the fundamental purpose of advertising sundry articles of merchandise to the use of dentists. It is inconceivable that in such journals anything should be published which would reflect upon the reputation or the quality of goods offered by the manufacturing house which publishes the trade journal. To be sure, conflicts may seldom arise; but if they arise once in a score of years, that single occasion must to a scientific mind forever condemn a trade journal. What we most desire is to establish the truth, and when established to publish it to the world. If it is possible that a truth being established, its publication (because of its conflict with trade interests) is withheld by a trade journal, how humiliating to the discoverer! And yet this is what may happen at any time if we rely on trade journals,—journals representing manufacturing companies whose interests are built upon patents, and whose desire is to have as many of their own manufactured articles used as possible. Evidently we have run across a divided interest, and as money-making is the main object of every business concern, when there is a conflict between the dentist's professional well-being and the success of a manufactured article, there is little doubt as to which interest will be uppermost. After all, one reason why we so readily allow our journalism to be managed for us is because it is so comfortable to be taken care of. In our southern country years ago it is probable that slavery in its best form was not altogether unpleasant to the slave; a kind master looked out very carefully for his welfare, and he was happy and did not realize his bondage. A well-known historical writer says,[1] "The relations between master and slave in Virginia were so pleasant that the offer of freedom fell upon dull, uninterested ears. With light work and generous fare, the condition of the Virginia negro was a happy one. The time had not yet come when he was liable to be torn from wife and children to die of hardship in the cotton-field and rice-swamps of the far South. He was proud of his connection with his master's estate and family, and had nothing to gain by rebellion."

That to my mind represents the condition of the body of the dental profession to-day as far as journalism is concerned. They are under an agreeable and apparently advantageous bondage to the manufacturing houses. Care is taken that this bondage be not too apparent; liberty of thought and action is assured along many lines; it is only when the line which separates the interests of the profession and the interests of business is approached that a halt is called, and it is evident that there are some paths which may not be travelled in a trade journal.

It is a fair question to ask, Why should a writer upon dental subjects prefer to publish his work in a trade journal rather than

---

[1] John Fiske, "The American Revolution," vol. i. p. 178.

in a professional journal? As far as I can determine, the reasons are these: A trade journal, owing to its business capital, is often able to pay for its articles; a professional journal seldom can do this. Secondly, a trade journal, if it does not pay directly for articles, may offer to illustrate them to the satisfaction of the author and without expense. This is a great inducement, and one which the professional journal may be unable to offer. Thirdly, a trade journal may be able to give an article a wider circulation than a professional journal. These three reasons why many prefer a trade journal for the publication of their articles, are strong ones; they appeal to a writer's pocket and to his self-importance or vanity.

It takes a good deal of courage for one to give time and perhaps money to the preparation of an article, and then have it appear without suitable illustrations, and in a journal which, though it may have a good circulation, does not have the circulation which a trade journal may control. I am reminded in this connection of the action of the Harvard Odontological Society some ten years ago, when it first decided to publish its transactions. Its first thought was to find a thoroughly professional journal in which work could be published. It passed by all trade journals which would have been glad to have taken its proceedings, and have furnished ample illustrations and lightened many of the burdens of publication. It hired and paid for its own stenographer, and found a journal published west of the Mississippi whose circulation was known to be meagre, but which was a strictly professional journal. And in this journal it published all its transactions for a year or more. The Harvard Odontological Society is composed of graduates of the Harvard Dental School, and has always been an important society. Its members practically bind themselves to take their turn by lot in supplying papers. Now, this society, notwithstanding the importance and variety of its work, was more interested that the record of its work should be made in a thoroughly professional journal than that the record should be expensively illustrated or should have the maximum circulation.

It was deemed inconsistent that a society composed of graduates from an ancient university should express itself except according to methods prevalent in other departments of the university. Who would think of looking in an electrical supply manufacturing journal for published results of work done in one of the great electrical laboratories of the university? And why, then, should the Harvard Odontological Society make use of a trade journal as its organ? Considerations of this sort, which naturally occur to men in contact with the methods of scientific men, led the Harvard Odontological Society to select the remote and in many ways unimportant journal for its use. Later, when a more important professional journal was started nearer home, the society transferred its proceedings to its columns.

Let the spirit which this society has shown be more common, let individuals refuse to publish their papers in any journal except a professional one, and the prosperity of the professional journals will be assured. They will then be able to offer satisfactory illustrations, pay for articles, and provide the maximum circulation. It is common to hear men say, "Let both kinds of journals exist. Whichever is the better journal, I will subscribe for and use for my articles. If the professional journal does not equal the trade journal, it must take the consequences in diminished circulation." All this is said with the air of one who feels not the slightest responsibility whether one kind of journal or another flourishes.

There is rarely a disposition shown to make a personal sacrifice for the purpose of strengthening the hands of those who are laboring to publish the records of the profession solely for its own interests and free from commercial complications. Little account is taken of the fact that it is difficult for a journal without capital to compete in many ways with one which has. That only by enthusiastic support can the professional journal be what all desire it to be. This enthusiastic support may be made to more than atone for large capital.

The action of the British Dental Association (referred to above) in setting aside one-third of its annual fee for the expense of the publication of the *Journal of the British Dental Association* cannot be too highly commended. In this way a journal can be placed upon a substantial financial basis, and thus be free from the temptation to bid for pecuniary success at the expense of principle. Why would it not be possible for some of our American societies to contribute a fixed part of the annual dues for the purpose of putting on a firm financial basis a journal of which we approve? Professional journalism can only be advanced by the development of a thorough professional spirit. Trade journals can only appeal to one whose professional sense is lowered by the spirit of trade. The strength of trade journals is in inverse proportion to the strength of the profession.

